# Bioactive Spirulina Protein Hydrolysate Mitigates Cyclophosphamide‐Induced Physiological, Biochemical, Histopathological, and Oxidative Stress Alterations in Rats

**DOI:** 10.1155/sci5/3555590

**Published:** 2026-11-04

**Authors:** Kittiya Phinyo, Pachara Sattayawat, Jeeraporn Pekkoh, Kritsana Duangjan, Supap Saenphet, Phongsadathon Aonmanee, Sirasit Srinuanpan, Khomsan Ruangrit, Chayakorn Pumas, Wararut Buncharoen

**Affiliations:** ^1^ Office of Research Administration Office of the University Chiang Mai University Chiang Mai 50200 Thailand cmu.ac.th; ^2^ Department of Biology Faculty of Science Chiang Mai University Chiang Mai 50200 Thailand cmu.ac.th; ^3^ Multidisciplinary Research Institute Chiang Mai University Chiang Mai 50200 Thailand cmu.ac.th

**Keywords:** blood biochemistry, cyclophosphamide, histopathology, protein hydrolysate, spirulina

## Abstract

Chemotherapy‐induced toxicity remains a significant challenge in cancer treatment; natural product‐derived dietary supplements have attracted considerable attention as potential protective agents. Spirulina, a nutrient‐rich filamentous cyanobacterium widely used as a dietary supplement, is recognized for its antioxidant and anti‐inflammatory properties. Here, spirulina protein hydrolysate was evaluated for its protective effects against cyclophosphamide‐induced systemic toxicity in male Wistar rats aged 8 weeks at arrival. Following 1 week of acclimatization, rats were allocated to four groups (*n* = 6/group): normal control, cyclophosphamide control, and two hydrolysate‐treated groups. The hydrolysate was administered orally at 3 or 6 mg/kg body weight daily for 14 days, with cyclophosphamide (200 mg/kg body weight) injected intraperitoneally on Days 7 and 14. Histopathological examination used hematoxylin and eosin staining. Cyclophosphamide exposure was associated with lower body weight and sperm viability, whereas hydrolysate‐treated rats continued to gain weight and exhibited higher sperm viability than cyclophosphamide controls (*p* < 0.05). Hydrolysate supplementation shifted selected serum biochemical parameters toward normal‐control values. Serum albumin concentrations were 2.52 ± 0.18 g/dL in cyclophosphamide controls and 3.62 ± 0.23 and 3.63 ± 0.37 g/dL in the 3 and 6 mg/kg hydrolysate groups, respectively (mean ± SD; both *p* < 0.05 vs. cyclophosphamide controls). Histopathological examination revealed cyclophosphamide‐associated damage in the liver, kidney, lung, testis, epididymis, seminal vesicle, and prostate gland. Hydrolysate‐treated groups, particularly the high‐dose group, showed better preservation of tissue architecture and fewer inflammatory and degenerative changes. These findings suggest that spirulina protein hydrolysate may mitigate selected cyclophosphamide‐associated physiological, biochemical, and histopathological alterations, providing preliminary preclinical evidence for its potential as a supportive dietary intervention.

## 1. Introduction

Spirulina is a well‐known cyanobacterium widely used as a nutritional supplement due to its diverse bioactive compounds and rich protein content [1]. The extracts of spirulina have attracted increasing attention as a functional ingredient, particularly when considering that the extracts may allow for more available bioactive compounds compared with the whole biomass. Spirulina can be processed into several types of extracts, including aqueous extracts, polysaccharide extracts, phycocyanin extracts, and protein hydrolysates, each containing different bioactive compounds with diverse biological activities [2]. Spirulina aqueous and phycocyanin extracts are well‐known for their antioxidant activity [3, 4]. Spirulina polysaccharide extract was recently reported to improve the immune cell killing ability against cholangiocarcinoma cell lines [5]. Among these preparations, spirulina protein hydrolysates (SPHs) are of particular interest because enzymatic hydrolysis provides a means of generating peptide‐containing preparations from the protein‐rich biomass. Previous studies have reported antioxidant activity [6], in vitro immunomodulatory activity, including enhanced phagocytic activity of polymorphonuclear leucocytes [7], and protection against dexamethasone‐induced atrophy in cultured myotubes [8]. These findings provide a rationale for investigating whether a SPH can influence oxidative stress‐related and tissue‐level outcomes during cyclophosphamide (CP) exposure. However, biological activities demonstrated in other experimental systems do not establish protection against CP‐induced multiorgan toxicity, and the contribution of specific peptide components remains to be determined.

CP is an alkylating agent widely used for the treatment of various cancers. Specifically, this chemotherapeutic drug has been applied in the treatment of ovarian adenocarcinoma, breast adenocarcinoma, small cell lung carcinoma, pancreatic ductal adenocarcinoma, and neuroblastoma [9]. However, despite its therapeutic effectiveness, CP is associated with several adverse effects, including nephrotoxicity, cardiotoxicity, reproductive toxicity, and hepatotoxicity, which may contribute to systemic organ dysfunction during chemotherapy treatment [10]. CP is a prodrug that undergoes hepatic cytochrome P450‐mediated activation to form 4‐hydroxycyclophosphamide, which exists in equilibrium with aldophosphamide and subsequently decomposes into phosphoramide mustard and acrolein [11]. Phosphoramide mustard mediates the antineoplastic effect of CP by inducing DNA alkylation and forming DNA interstrand cross‐links, lesions that are closely linked to cytotoxic activity [12, 13]. In contrast, acrolein is a highly reactive electrophilic metabolite that significantly contributes to off‐target tissue toxicity [14, 15]. Acrolein promotes excessive reactive oxygen species (ROS) production, disrupts mitochondrial function, and reacts with cellular sulfhydryl‐containing molecules, thereby disturbing cellular redox homeostasis and antioxidant capacity [15, 16]. Excessive ROS formation can increase lipid peroxidation and deplete or suppress endogenous antioxidant defenses, including reduced glutathione (GSH) and the antioxidant enzymes, superoxide dismutase (SOD), catalase (CAT), glutathione peroxidase (GPx), glutathione reductase (GR), and glutathione‐*S*‐transferase (GST) [17]. Consequently, CP‐induced oxidative stress can promote lipid, protein, and DNA damage, as well as mitochondrial dysfunction, inflammation, apoptosis, cellular degeneration, and histopathological injury [16, 18]. These oxidative and pathological effects have been documented in several organs and tissues, including the liver, kidneys, urinary bladder, lungs, heart, testes, and other reproductive tissues [15, 17–21], suggesting that CP induces systemic toxicity and can cause multiorgan damage beyond its intended therapeutic effects. However, the severity and distribution of CP‐induced injury may vary according to the administered dose, dosing schedule, exposure duration, sampling time, animal model, and tissue susceptibility [18, 20].

Previous research provides evidence that whole spirulina biomass can ameliorate CP‐associated reproductive injury. Afkhami‐Ardakani et al. [22] reported that oral *Spirulina platensis* supplementation at 500 and 1000 mg/kg/day for 4 weeks improved epididymal sperm counts and testicular histomorphometric indices and lowered serum malondialdehyde (MDA) concentrations in CP‐treated male Wistar rats. However, that study evaluated whole spirulina powder and focused on testicular injury. Its findings therefore do not establish whether an enzymatically prepared protein hydrolysate can influence CP‐associated alterations across multiple organs and physiological systems. Likewise, the biological activities reported for other spirulina extracts cannot be assumed to apply to protein hydrolysates because these preparations differ in composition.

The hypothesis that oral SPH administration would attenuate selected CP‐associated physiological, reproductive, biochemical, oxidative stress, and histopathological alterations in male Wistar rats. The present study therefore investigated SPH at 3 and 6 mg/kg body weight (BW). These properties suggest that SPH may be beneficial as a supportive dietary supplement for reducing CP‐associated systemic toxicity; however, further investigations are required. Therefore, this study aims to evaluate the effects of SPH on physiological parameters, blood biochemical markers, histopathological alterations of internal organs, and serum oxidative stress alterations as a food supplement for CP‐induced rats to determine its potential to attenuate systemic toxicity.

## 2. Materials and Methods

### 2.1. SPH

SPH was prepared at the Algae and Cyanobacteria Research Laboratory, Department of Biology, Faculty of Science, Chiang Mai University, Thailand. Briefly, dried spirulina biomass (Li Algae Biotech Company Limited, Thailand) was pretreated with 90% methanol to remove chlorophyll, followed by enzymatic protein extraction using Viscozyme (Novozymes, Bagsvaerd, Denmark) combined with ultrasonic assistance, yielding approximately 37.50% (w/w) of freeze‐dried protein extract relative to the initial dry biomass. The extracted protein was subsequently hydrolyzed with Alcalase under ultrasonic assistance using optimized conditions established in preliminary experiments by response surface methodology based on a central composite design, following the experimental approach of Pekkoh et al. [23]. The optimized conditions were pH 6.93, 58.03°C, 56.94 min, and an Alcalase‐to‐protein ratio of 4.48% (w/w). Following hydrolysis, the enzyme was inactivated at 90°C for 20 min, and the hydrolysate was centrifuged at 6000 rpm for 20 min at 4°C, freeze‐dried for 48 h, and stored at −20°C until use.

### 2.2. Animals and Experimental Design

#### 2.2.1. Animals

Twenty‐four male Wistar rats (*Rattus norvegicus*) at the age of 8 weeks and with the BW of 250–300 g were obtained from Nomura Siam International Co., Ltd., Thailand. The rats were acclimated under controlled laboratory circumstances (25 ± 1°C) for one week. Rats were housed in two animals per cage in cages measuring 24 × 40 × 18 cm with corn cob bedding, with three cages per treatment group. During the experimental period, commercial pelleted rat food (SmartHeart, Thailand) and water were administered *ad libitum*.

#### 2.2.2. Ethical Considerations

All experimental techniques using animals were performed in compliance with the rules for the care and use of laboratory animals and authorized by the Animal Ethics Committee, Department of Biology, Faculty of Science, Chiang Mai University (Approval No. Re 001/23). Animal health and welfare were examined daily during the trial period for any changes in overall appearance, behavior, food and water consumption, BW, and clinical symptoms of discomfort or ill effects. Predefined humane endpoints were a ≥ 20% decrease in BW compared with the pretreatment baseline, no food intake for 48 consecutive hours, hematuria, chronic stooped posture, and severe diarrhea. Any animal that fit into any of these categories had to be humanely euthanized immediately. None of the animals attained the predetermined humane endpoints, and no death was observed during the trial period.

#### 2.2.3. Experimental Design

After acclimatization, animals were stratified according to baseline BW to minimize disparities in initial BW among experimental groups. Animals within each body‐weight stratum were randomly allocated to one of four experimental groups (*n* = 6 rats/group) using a random‐number method: normal control (NC), CP‐treated control (CP), and SPH‐treated groups (SPH3 and SPH6) receiving 3 and 6 mg/kg BW, respectively. The number of animals used in each group was determined based on prior studies using similar models of CP toxicity [24]. The experimental unit for outcomes measured at the individual level was the individual animal (*n* = 6 rats per group), but for food and water intake, which were measured at the cage level as a whole, the experimental unit was the cage (*n* = 3 cages per group). SPH was delivered orally once daily for 14 days. Systemic toxicity was induced by intraperitoneal administration of CP (200 mg/kg BW) on Days 7 and 14. The experimental setup and schedule of treatment are shown in Figure 1. On Day 15, animals were extensively anesthetized with isoflurane, and final blood was collected by cardiac puncture. Then biological samples and organs were taken for biochemical and histological analysis.

**FIGURE 1:**
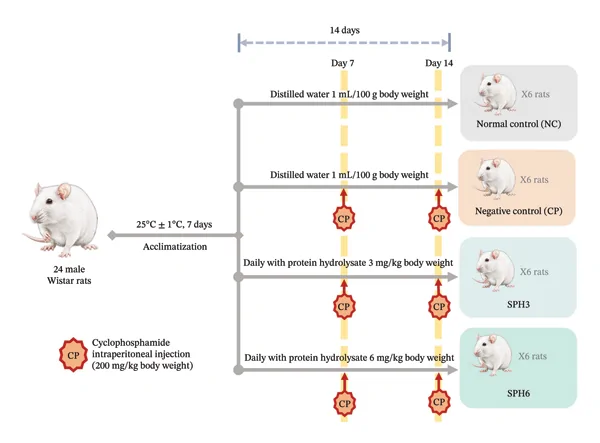
Experimental design of cyclophosphamide‐induced toxicity and spirulina protein hydrolysate treatment in male Wistar rats. Rats were assigned to four groups (*n* = 6 per group): normal control (NC), cyclophosphamide‐treated control (CP), SPH3, and SPH6. Distilled water or spirulina protein hydrolysate (3 or 6 mg/kg body weight) was administered orally once daily for 14 days. Cyclophosphamide (200 mg/kg body weight, intraperitoneally) was administered on Days 7 and 14 to all groups except NC.

### 2.3. BW, Food Intake, and Water Intake

BW was measured individually at baseline and 7‐day intervals thereafter using a digital balance (AB204‐S Mettler‐Toledo, Switzerland, with a maximal capacity of 220 g). Food and water consumption were determined at 7‐day intervals over a 24‐h measurement period. Each treatment group consisted of six rats housed two per cage, resulting in three cages per group. For food intake measurements, a known amount of feed was provided in a designated food compartment physically separated from the bedding area. At the end of each 24‐h measurement period, residual feed remaining in the food compartment was collected and weighed. Feed particles spilled onto the corn cob bedding were carefully recovered, separated from the bedding materials, and combined with the residual feed before weighing. Food intake per cage was calculated as the following formula:

(1) food intake g=feed provided g–total feed recovered g,

where total feed recovered included both residual feed remaining in the food compartment and feed recovered from the bedding.

Water intake was measured during the same 24‐h period by recording the initial volume of water provided in each cage and the volume remaining after 24 h. Water bottles were inspected for leakage before use. Water intake was calculated separately for each cage as the following formula:

(2) water intake mL=initial water volume mL–remaining water volume mL.

Because food and water intake were measured collectively for the two rats housed in each cage, the cage was considered the experimental unit for these parameters. Thus, each treatment group provided three independent cage‐level observations (*n* = 3 cages/group), which were used for statistical comparisons among treatment groups. BW, in contrast, was measured individually, with the individual rat considered the experimental unit (*n* = 6 rats/group).

### 2.4. Relative Organ Weights

At the end of the experimental period, rats were euthanized, and internal organs, including liver, kidneys, lungs, and hearts, and reproductive organs (testes, epididymis, seminal vesicles, and prostate glands) were carefully collected, rinsed with normal saline, and trimmed to remove surrounding adipose tissues before weighing. The organs were excised, cleaned of adhering tissues, and weighed immediately using an analytical balance (AB204‐S Mettler‐Toledo, Switzerland, with a maximal capacity of 220 g). Relative organ weights were calculated by normalizing organ weight to the final BW of each animal according to the following formula:

(3) relative organ weight g/100 g BW=organ weightBW×100.

### 2.5. Assessment of Sperm Quality

The cauda epididymis of each rat was carefully excised and placed into a glass vial containing 1 mL of prewarmed Krebs–Henseleit solution (NaCl 118.1 mM, KH_2_PO_4_ 1.2 mM, KCl 4.7 mM, NaHCO_3_ 25.0 mM, glucose (GLU) 11.7 mM, MgSO_4_ 0.5 mM, and CaCl_2_ 2.5 mM, pH 7.4) maintained at 37°C. The cauda epididymis was minced finely until the suspension became visibly turbid in order to release spermatozoa. A further 9 mL of Krebs–Henseleit solution was then added to obtain the sperm suspension. The sample was then utilized to evaluate sperm motility, concentration, viability, and morphology. The sperm suspension was maintained at 37°C during handling and analyzed within 15 min of preparation.

#### 2.5.1. Sperm Morphology

For sperm morphology, an aliquot of 10 μL of sperm suspension was dropped on a clean glass slide and smeared (3 slides per rat). The slides were air‐dried, fixed in methanol, stained with Wright–Giemsa, and analyzed at 40x magnification. The abnormalities in spermatozoa head and tail (200 spermatozoa per slide) were evaluated [25]. A total of 200 sperm cells per sample were evaluated and classified into four morphological categories: normal morphology, head abnormality, tail abnormality, and combined head and tail abnormalities. The percentage of each morphological category was calculated as follows:

(4) morphological category %=number of spermatozoa in each morphological categorytotal number of spermatozoa counted×100.

#### 2.5.2. Sperm Concentration

Prior to hemocytometer counting, one part of the epididymal sperm suspension was mixed with 20 parts of 10% neutral buffered formalin to immobilize the spermatozoa. Subsequently, 10 μL of the fixed sperm suspension from each rat was loaded into a Neubauer hemocytometer and allowed to stand for 5 min to permit the sperm cells to settle. The spermatozoa were then examined under a light microscope using a 40× objective lens and counted in nine small squares of the hemocytometer grid. Sperm counting was performed in triplicate for each rat. Sperm concentration was calculated based on the total number of spermatozoa counted, the volume of the hematocytometer chamber, and the dilution factor using the following formula:

(5) sperm concentration cellsmL=total number of spermatozoa counted×dilution factorcounted chamber volume mL.

#### 2.5.3. Sperm Viability

Sperm viability was assessed by the trypan blue exclusion method [26]. In brief, 10 μL of epididymal sperm suspension was mixed with an equal volume of 0.4% trypan blue. The mixture was incubated for 3 min. After incubation, a 10 μL sample of sperm was placed on a microscopic slide. The viability of sperm was assessed using a phase contrast microscope at 100x magnification. The number of spermatozoa counted was up to 200 per slide. Unstained spermatozoa were classified as viable, whereas blue‐stained spermatozoa were classified as nonviable. Sperm viability was determined in each rat in triplicate. Sperm viability was expressed as the percentage of viable spermatozoa relative to the total number of spermatozoa counted as the following formula:

(6) sperm viability %=number of viable spermatozoatotal number of spermatozoa counted×100.

#### 2.5.4. Sperm Motility

Sperm motility was determined by placing 10 μL of epididymal sperm suspension on a clean glass slide and covering it with a coverslip. Sperm motility was observed by a phase contrast microscope with 20x magnification. The pattern of sperm movement was recorded for 200 spermatozoa per slide, which was classified as progressive movement, nonprogressive movement, circular movement, or nonmovement (immotile sperm) [26]. Sperm motility of each rat was determined in triplicate. Finally, a percentage of each movement was calculated as follows:

(7) sperm in each motility category %=number of spermatozoa in each motility categorytotal number of spermatozoa counted×100.

### 2.6. Biochemical Analysis

Following the treatment period, blood samples were collected via cardiac puncture under anesthesia into non‐anticoagulant tubes and centrifuged at 3500 rpm for 10 min to isolate serum. The serum was used for biochemical analyses, and aliquots for oxidative stress and antioxidant assays were stored at −20°C until analysis.

#### 2.6.1. Serum Biochemical Parameters

Serum biochemical parameters were analyzed using a Chemistry Analyzer MS100 (Zhejiang Pushkang Biotechnology Co., Ltd., China) with the Veterinary General Chemistry 23 Test Panel according to the manufacturer’s instructions. The parameters analyzed included total protein (TP), albumin (ALB), globulin (GLO), albumin/globulin ratio (A/G), alanine aminotransferase (ALT), aspartate aminotransferase (AST), alkaline phosphatase (ALP), gamma‐glutamyl transferase (GGT), total bilirubin (TBIL), direct bilirubin (DBIL), indirect bilirubin (IBIL), total bile acids (TBA), AST/ALT ratio, blood urea nitrogen (BUN), creatinine (CRE), BUN/CRE ratio, GLU, total cholesterol (TCH), amylase (AMY), calcium (Ca), phosphorus (PHOS), carbon dioxide (CO_2_), and creatine kinase (CK). Each biological serum sample was analyzed once without technical replication. The automated analyzer was operated using the manufacturer‐specified lyophilized reagent discs and automatic reagent identification system according to the manufacturer’s procedures. Results were expressed as g/dL for TP, ALB, and GLO; U/L for ALT, AST, ALP, GGT, AMY, and CK; mg/dL for TBIL, DBIL, IBIL, BUN, CRE, GLU, TCH, and Ca; μmol/L for TBA; and mmol/L for PHOS and CO_2_. The A/G, AST/ALT, and BUN/CRE ratios were dimensionless.

#### 2.6.2. Serum Oxidative Stress Biomarkers

Serum MDA content was determined using the thiobarbituric acid‐reactive substances assay at 532 nm, with tetramethoxypropane as the standard [27, 28]. GSH content was determined following deproteinization with 4% (w/v) sulfosalicylic acid and reaction with 5,5′‐dithiobis‐(2‐nitrobenzoic acid) at 412 nm [29]. CAT activity was assessed by monitoring hydrogen peroxide decomposition at 240 nm [30], whereas SOD activity was determined using the xanthine–xanthine oxidase/nitroblue tetrazolium system at 560 nm [28, 31]. GST activity was determined using GSH and 1‐chloro‐2,4‐dinitrobenzene at 340 nm [32]. GR and GPx activities were determined by monitoring NADPH oxidation at 340 nm [33, 34].

Each rat represented one biological replicate, and one serum sample obtained from each rat was used for each oxidative stress assay. Each serum sample was assayed once without technical replication. Levels of MDA, SOD, CAT, and GSH were calculated from their respective standard curves. The calibration equations used for MDA, SOD, CAT, and GSH were *y* = 0.0003*x* + 0.0107, *y* = 0.00005*x* − 0.0011, *y* = 0.0009*x* − 0.0007, and *y* = 0.0003*x* + 0.0259, respectively. GST, GR, and GPx activities were calculated from the rate of change in absorbance using molar extinction coefficients of 9.6 × 10^3^, 6.22 × 10^3^, and 6.22 × 10^3^ M^−1 ^cm^−1^, respectively. The calculated enzyme activities were subsequently normalized to the protein content of the corresponding samples. Results were expressed as nmol/mg protein for MDA, mmol/mg protein for GSH, U/mg protein for SOD and CAT, μmol CDNB conjugate formed/min/mg protein for GST, and nmol NADPH oxidized/min/mg protein for GR and GPx.

### 2.7. Histopathological Observations

For histopathological examination, the liver, kidneys, lungs, heart, and reproductive organs of each rat were fixed in 10% (v/v) neutral buffered formalin for 24 h. For reproductive tissue histopathology, the cauda epididymis and ventral lobe of the prostate gland were consistently sampled and examined across all experimental groups. The tissues were dehydrated through ascending concentrations of ethanol, cleared in xylene, infiltrated with molten paraffin, and embedded in paraffin blocks. Paraffin‐embedded tissues were sectioned at 6 μm using a rotary microtome, mounted on glass slides, and dried on a slide warmer. Histological sections were stained with hematoxylin and eosin (H&E). Briefly, the sections were deparaffinized in xylene for 5 min (two changes) and rehydrated through a graded ethanol series (100%, 95%, 85%, and 70%), followed by distilled water, with each step performed for 5 min. The sections were stained with Harris’ hematoxylin for 5 min and washed under running tap water for 10 min. Excess hematoxylin was differentiated with 1% acid alcohol, followed by treatment with 0.2% ammonia water. The sections were then counterstained with eosin for 3 min, dehydrated through ascending concentrations of ethanol (70%, 85%, 95%, and 100%), cleared in xylene, and mounted with Permount mounting medium and coverslips prior to microscopic examination. Histopathological evaluation was performed by an experienced pathologist who was blinded to treatment allocation. Before evaluation, all tissue slides were assigned numerical codes that concealed the treatment‐group identities from the evaluator. The treatment codes were revealed only after completion of the histopathological assessment. Histopathological examination was performed at multiple magnifications (40x–400x), with lower magnifications used to assess overall tissue architecture and lesion distribution and higher magnifications used to evaluate cellular alterations.

### 2.8. Data Analysis

Statistical analyses were performed using PAST Version 4.03 and R Version 4.6.1. Data are presented as mean ± standard deviation (SD). Normality was assessed using the Shapiro–Wilk test, and homogeneity of variances was evaluated using Levene’s test. Normally distributed data satisfying the homogeneity of variance assumption were analyzed using one‐way analysis of variance (ANOVA), followed by Tukey’s pairwise comparisons. When normality was satisfied but homogeneity of variance was violated, Welch’s ANOVA was applied, followed by the Games–Howell post hoc test using the rstatix package in R. Nonnormally distributed data were analyzed using the Kruskal–Wallis test, followed by Dunn’s post hoc test. Differences were considered statistically significant at *p* < 0.05.

## 3. Results

### 3.1. Effects of SPH on Physiological Parameters of Rats Induced With CP

#### 3.1.1. BW and Food–Water Intake

BW did not differ significantly among the experimental groups on Day 1 (Kruskal–Wallis test, *p* = 0.206), whereas a significant difference was detected on Day 7 (*p* = 0.009). By Day 14, BW differed markedly among groups (one‐way ANOVA, *p* < 0.001). The CP group had significantly lower BW than the NC, SPH6, and SPH3 groups, whereas the two SPH‐treated groups did not differ significantly from each other. Mean BWs on Day 14 were 386.67 ± 6.06, 260.83 ± 17.44, 334.17 ± 15.30, and 348.33 ± 27.33 g in the NC, CP, SPH6, and SPH3 groups, respectively (Figure 2A).

**FIGURE 2:**
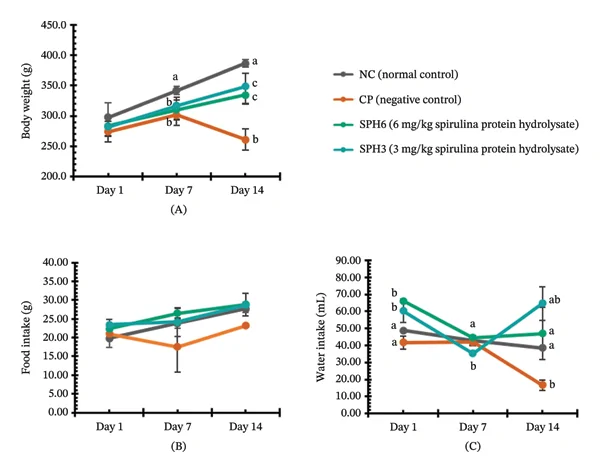
Effects of spirulina protein hydrolysate on physiological parameters: (A) body weight, (B) food intake, and (C) water intake in cyclophosphamide‐treated rats compared with the normal control. Data are presented as mean ± standard deviation (SD). For body weight (A), individual rats were considered the experimental units (*n* = 6 rats per group). For food intake (B) and water intake (C), cages were considered the experimental units (*n* = 3 cages per group, with two rats per cage). Different letters indicate statistically significant differences among treatment groups within each parameter at the same time point (*p* < 0.05), whereas groups sharing at least one letter are not significantly different.

Food intake did not differ significantly among groups on Days 1, 7, or 14 (*p* > 0.05), although the CP group showed numerically lower mean intake on Days 7 and 14 (Figure 2B). In contrast, water intake differed significantly among groups at the evaluated time points. On Day 14, a significant group difference in water intake was observed (Kruskal–Wallis test, *p* = 0.039), with the CP group showing the lowest mean value (16.67 ± 3.06 mL/cage).

#### 3.1.2. Relative Organ Weights

The relative weights of the liver, kidney, lung, and heart are presented in Figure 3. No significant differences in relative liver weight were observed among the NC, CP, SPH6, and SPH3 groups (*p* = 0.384; Figure 3A). Relative kidney weight differed significantly among the experimental groups (*p* < 0.001), with the CP group showing a significantly higher value than the NC group (Figure 3B). Relative lung weight also differed significantly among groups (*p* < 0.001); the CP group had significantly higher values than the NC, SPH3, and SPH6 groups, while the SPH6 group also had a significantly higher value than the NC group (Figure 3C). For the heart, a significant overall difference was observed among groups (*p* = 0.012), with the SPH3 group showing a significantly higher relative heart weight than the NC group (Figure 3D).

**FIGURE 3:**
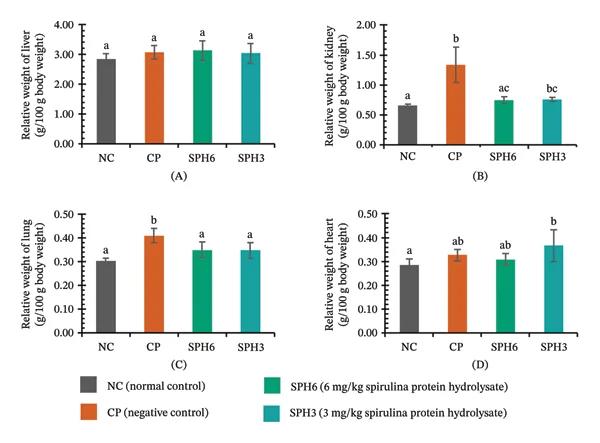
Relative organ weights in cyclophosphamide‐treated rats receiving spirulina protein hydrolysate: (A) liver, (B) kidney, (C) lung, and (D) heart. Data are presented as mean ± standard deviation (SD) of six rats per group. Liver, lung, and heart data were analyzed using one‐way ANOVA followed by Tukey’s pairwise comparisons, whereas kidney data were analyzed using the Kruskal–Wallis test followed by Dunn’s post hoc test. Different letters indicate significant differences among groups within each organ (*p* < 0.05), whereas shared letters indicate no significant difference.

Regarding the reproductive organs, relative testes weight differed significantly among the experimental groups (one‐way ANOVA, *p* < 0.001). The CP group had a significantly higher relative testes weight than the NC (*p* < 0.001), SPH6 (*p* = 0.035), and SPH3 (*p* = 0.005) groups, whereas the SPH6 and SPH3 groups did not differ significantly from the NC group (Table 1). Relative epididymis weight also differed significantly among groups (Kruskal–Wallis test, *p* = 0.022). Dunn’s post hoc test showed a significant difference between the CP and NC groups (*p* = 0.002), whereas no significant differences were detected among the other pairwise comparisons. Relative prostate gland and seminal vesicle weights did not differ significantly among the experimental groups (*p* = 0.459 and *p* = 0.238, respectively).

**TABLE 1:** Relative reproductive organ weights in cyclophosphamide‐induced rats treated with spirulina protein hydrolysate.

Experimental set	Relative weight of reproductive organ			
	Testes (g/100 g BW)	Epididymis (g/100 g BW)	Prostate gland (g/100 g BW)	Seminal vesicle (g/100 g BW)
NC	0.43 ± 0.02^a^	0.13 ± 0.01^a^	0.14 ± 0.02^a^	0.28 ± 0.04^a^
CP	0.57 ± 0.05^b^	0.19 ± 0.02^b^	0.16 ± 0.03^a^	0.37 ± 0.16^a^
SPH6	0.49 ± 0.03^a^	0.15 ± 0.01^ab^	0.15 ± 0.01^a^	0.33 ± 0.09^a^
SPH3	0.46 ± 0.05^a^	0.16 ± 0.03^ab^	0.21 ± 0.07^a^	0.23 ± 0.04^a^

*Note:* Values are presented as mean ± standard deviation (SD) of six rats per group (*n* = 6) and expressed as g/100 g body weight (BW). Testes data were analyzed using one‐way ANOVA followed by Tukey’s pairwise comparisons. Epididymis and prostate gland data were analyzed using the Kruskal–Wallis test, with Dunn’s post hoc test applied to epididymis data following a significant overall difference. Seminal vesicle data were analyzed using one‐way ANOVA. Different superscript letters within the same column indicate significant differences among groups, whereas values sharing at least one letter are not significantly different (*p* < 0.05).

### 3.2. Assessment of Sperm Quality

#### 3.2.1. Sperm Morphology

Normal sperm morphology was characterized by a curved head and a single uncoiled tail (Figure 4A). Morphological abnormalities involving the head, tail, or both head and tail were observed across the experimental groups (Figure 4B–E). The proportions of morphologically normal sperm were 64.13%, 56.83%, 59.75%, and 57.50% in the NC, CP, SPH6, and SPH3 groups, respectively. Tail abnormalities were the predominant morphological abnormality, accounting for 35.25%, 41.33%, 39.38%, and 41.88%, respectively. Head abnormalities accounted for 0.63%, 1.83%, 0.63%, and 0.38%, respectively, whereas combined head‐and‐tail abnormalities accounted for 0%, 0%, 0.25%, and 0.25%, respectively (Figure 5C).

**FIGURE 4:**
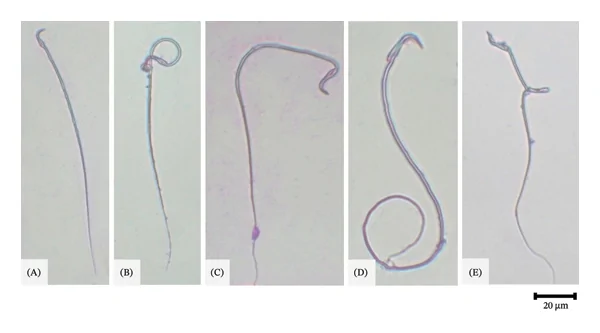
Sperm morphology of cyclophosphamide‐induced rats treated with spirulina protein hydrolysate. (A) Normal sperm morphology; (B–C) spermatozoa with head abnormalities; (D) spermatozoon with a tail abnormality; and (E) spermatozoon with combined head‐and‐tail abnormalities. Sperm smears were stained with Wright–Giemsa and examined under a light microscope at 40x magnification. Scale bar = 20 μm.

**FIGURE 5:**
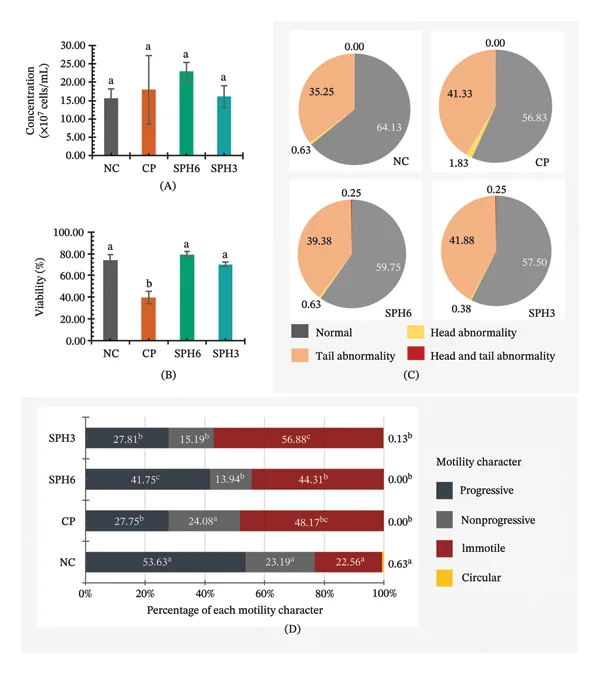
Assessment of sperm quality in cyclophosphamide‐treated rats receiving spirulina protein hydrolysate. (A) Sperm concentration, (B) sperm viability, (C) distribution of normal and morphologically abnormal sperm, and (D) sperm motility patterns, including progressive, nonprogressive, immotile, and circular movement. Data are presented as mean ± standard deviation (SD) of six rats per group (*n* = 6). Different letters indicate statistically significant differences among treatment groups within each sperm parameter (*p* < 0.05), whereas values sharing at least one letter are not significantly different.

#### 3.2.2. Sperm Concentration

Sperm concentration showed no significant differences among the experimental groups (Figure 5A).

#### 3.2.3. Sperm Viability

Sperm viability was significantly lower in the CP group than in the NC group. In contrast, the SPH6 and SPH3 groups showed significantly higher sperm viability than the CP group, with values comparable to those of the NC group (Figure 5B).

#### 3.2.4. Sperm Motility

Sperm motility patterns differed among the experimental groups (Figure 5D). The NC group showed the highest progressive motility and the lowest proportion of immotile sperm. In contrast, the CP group exhibited lower progressive motility and a higher proportion of immotile sperm than the NC group. The SPH6 group showed higher progressive motility than the CP group, although the value remained lower than that of the NC group. The SPH3 group showed progressive motility comparable to that of the CP group and a higher proportion of immotile sperm than the NC group. Circular movement was low across all experimental groups.

### 3.3. Biochemical Analyses

#### 3.3.1. Serum Biochemical Parameters

Serum biochemical parameters are presented in Table 2. Compared with the NC group, the CP group showed significantly lower ALB, ALB/GLB, CO_2_, AMY, and CK levels and significantly higher ALP, AST, TBA, BUN, and urea/creatinine ratio (*p* < 0.05). No significant differences between the CP and NC groups were observed for TP, ALT, AST/ALT, CRE, TCH, GGT, DBIL, TBIL, GLOB, IBIL, Ca, or GLU.

**TABLE 2:** Serum biochemical indices of cyclophosphamide‐induced rats treated with spirulina protein hydrolysate.

Parameter	NC	CP	SPH6	SPH3
ALB (g/dL)	3.57 ± 0.44^a^	2.52 ± 0.18^b^	3.63 ± 0.37^a^	3.62 ± 0.23^a^
TP (g/dL)	5.97 ± 0.60^ab^	5.13 ± 0.10^b^	6.55 ± 0.64^a^	6.40 ± 0.56^a^
ALP (U/L)	112.67 ± 13.89^a^	184.00 ± 18.00^b^	84.50 ± 7.20^c^	113.50 ± 15.39^a^
ALT (U/L)	21.67 ± 1.86^ab^	25.33 ± 2.07^b^	17.33 ± 3.78^a^	18.67 ± 3.27^a^
AST (U/L)	119.33 ± 17.93^a^	158.00 ± 12.82^b^	76.17 ± 7.86^c^	111.67 ± 9.61^a^
GGT (U/L)	4.17 ± 1.11^a^	3.68 ± 1.60^a^	3.62 ± 0.57^a^	3.57 ± 0.29^a^
DBIL (mg/dL)	0.08 ± 0.04^a^	0.07 ± 0.05^a^	0.08 ± 0.04^a^	0.07 ± 0.05^a^
TBIL (mg/dL)	0.25 ± 0.05^a^	0.27 ± 0.05^a^	0.20 ± 0.00^a^	0.25 ± 0.05^a^
GLOB (g/dL)	2.85 ± 0.43^a^	2.38 ± 0.28^a^	2.72 ± 0.33^a^	2.82 ± 0.37^a^
IBIL (mg/dL)	0.20 ± 0.00^a^	0.18 ± 0.04^a^	0.17 ± 0.05^a^	0.18 ± 0.04^a^
ALB/GLB	1.27 ± 0.18^a^	1.07 ± 0.13^b^	1.34 ± 0.08^a^	1.30 ± 0.14^a^
TBA (μmol/L)	4.47 ± 2.47^a^	7.42 ± 2.99^b^	4.17 ± 0.38^a^	3.80 ± 0.75^a^
AST/ALT	5.54 ± 0.95^ab^	6.30 ± 0.96^b^	4.53 ± 0.82^a^	6.15 ± 1.29^ab^
Ca (mg/dL)	8.86 ± 0.98^a^	8.72 ± 0.52^a^	8.89 ± 0.86^a^	9.01 ± 1.10^a^
Creatinine (mg/dL)	0.42 ± 0.04^a^	0.38 ± 0.04^ab^	0.35 ± 0.05^b^	0.33 ± 0.05^b^
CO_2_ (mmol/L)	22.93 ± 2.06^a^	13.33 ± 2.82^b^	23.68 ± 2.02^a^	23.67 ± 1.73^a^
BUN (mg/dL)	12.50 ± 1.87^a^	15.33 ± 0.52^b^	14.67 ± 1.51^bc^	13.17 ± 1.47^ac^
Urea/creatinine ratio	29.92 ± 2.13^a^	40.42 ± 4.85^b^	42.64 ± 6.90^b^	40.00 ± 5.16^b^
Total cholesterol (mg/dL)	66.64 ± 5.28^a^	57.27 ± 7.05^a^	82.82 ± 8.02^b^	78.45 ± 3.57^b^
Glucose (mg/dL)	193.10 ± 32.65^a^	187.08 ± 16.57^a^	156.36 ± 21.23^a^	197.30 ± 39.82^a^
Amylase (U/L)	1322.33 ± 127.61^a^	681.28 ± 158.11^b^	1246.33 ± 113.56^a^	1230.83 ± 144.69^a^
Creatine kinase (U/L)	257.47 ± 39.27^a^	166.20 ± 27.00^b^	274.08 ± 13.45^a^	288.58 ± 18.49^a^

*Note:* Values are the mean ± standard deviation (SD) of six rats per group. Different superscript letters in the same row indicate statistically significant differences among groups (*p* < 0.05), whereas values sharing at least one letter are not significantly different.

Compared with the CP group, both the SPH6 and SPH3 groups showed significantly higher ALB, TP, ALB/GLB, CO_2_, AMY, and CK levels and significantly lower ALP, ALT, AST, and TBA levels (*p* < 0.05). The AST/ALT ratio was significantly lower in the SPH6 group than in the CP group, whereas no significant difference was observed between the SPH3 and CP groups. BUN was significantly lower in the SPH3 group than in the CP group, while the SPH6 group did not differ significantly from the CP group. Neither SPH‐treated group differed significantly from the CP group in CRE or the urea/creatinine ratio. TCH was significantly higher in both SPH‐treated groups than in the CP and NC groups. Other parameters showing no significant differences among the experimental groups are detailed in Table 2.

#### 3.3.2. Serum Oxidative Stress Biomarkers

Serum oxidative stress and antioxidant parameters are presented in Figure 6. No significant differences in MDA concentration were observed among the experimental groups (Kruskal–Wallis test, *p* > 0.05) (Figure 6A). SOD activity differed significantly among groups (Welch’s ANOVA, *p* < 0.001). SOD activity was significantly lower in the SPH6 and SPH3 groups than in the NC group, whereas the CP group did not differ significantly from either the NC or SPH3 group. The SPH3 group showed significantly lower SOD activity than all other groups (*p* < 0.05) (Figure 6B).

**FIGURE 6:**
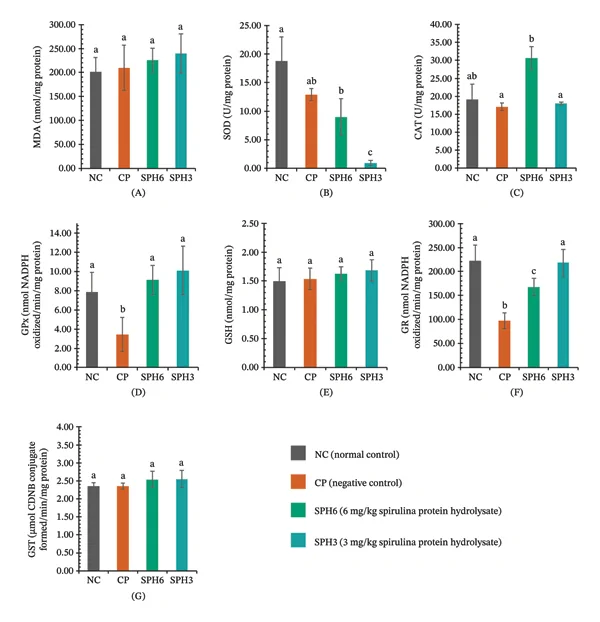
Effects of spirulina protein hydrolysate on serum oxidative stress and antioxidant status in cyclophosphamide‐treated Wistar rats. Serum levels or activities of (A) MDA: malondialdehyde, (B) SOD: superoxide dismutase, (C) CAT: catalase, (D) GPx: glutathione peroxidase, (E) GSH: reduced glutathione, (F) GR: glutathione reductase, and (G) GST: glutathione‐*S*‐transferase in rats from the NC, CP, SPH6, and SPH3 groups. Data are shown as mean ± standard deviation (SD) of six rats per group. Distinct letters denote statistically significant differences among treatment groups for each biomarker (*p* < 0.05).

CAT activity differed significantly among groups (one‐way ANOVA, *p* < 0.05). The SPH6 group showed significantly higher CAT activity than the CP and SPH3 groups, whereas no significant difference was observed between the SPH6 and NC groups (Figure 6C). GPx activity also differed significantly among groups (one‐way ANOVA, *p* < 0.001). The CP group showed significantly lower GPx activity than the NC, SPH6, and SPH3 groups, whereas no significant differences were observed among the NC and SPH‐treated groups (Figure 6D). GSH concentration did not differ significantly among the experimental groups (Kruskal–Wallis test, *p* > 0.05) (Figure 6E).

GR activity differed significantly among groups (one‐way ANOVA, *p* < 0.001). The CP group showed significantly lower GR activity than all other groups. GR activity in the SPH6 group was significantly higher than that in the CP group but significantly lower than those in the NC and SPH3 groups, whereas the SPH3 and NC groups did not differ significantly (Figure 6F). No significant differences in GST activity were observed among the experimental groups (Kruskal–Wallis test, *p* > 0.05) (Figure 6G).

### 3.4. Effects of SPH on Histopathological Alterations of Rats Induced With CP

Histopathological examination of liver tissues from the NC group showed preserved hepatic architecture, including well‐arranged hepatocytes, visible hepatic sinusoids, Kupffer cells, and intact vascular and portal structures (Figure 7A). In contrast, liver sections from the CP group showed hepatocellular necrosis, indicated by red arrows, together with disruption of normal hepatic organization (Figure 7B). In the SPH‐treated groups, representative sections showed generally preserved hepatocyte organization and hepatic sinusoids, as observed in the SPH6 (Figure 7C) and SPH3 (Figure 7D) groups.

**FIGURE 7:**
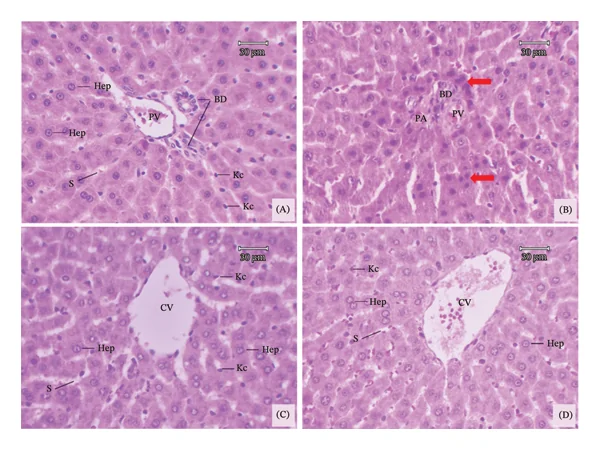
Histopathological features of hepatic tissues from cyclophosphamide‐treated rats receiving spirulina protein hydrolysate. Representative H&E‐stained liver sections from (A) NC, (B) CP, (C) SPH6, and (D) SPH3 groups. The panels illustrate representative hepatic regions containing portal and/or central venous structures, depending on the section examined. Red arrows indicate hepatocellular necrosis. Abbreviations: CV, central vein; PV, portal vein; BD, bile duct; PA, portal artery; Hep, hepatocytes; Kc, Kupffer cells; S, hepatic sinusoids; HC, hepatic cords. Scale bar = 30 μm.

Histopathological examination of renal tissues also revealed marked structural changes in the CP group. The NC group showed typical renal architecture, characterized by well‐preserved glomeruli, Bowman’s capsule, proximal convoluted tubules, and distal convoluted tubules (Figure 8A and B). In contrast, renal sections from the CP group exhibited dilated renal tubules, shrunken glomeruli, and inflammatory cell infiltration (Figure 8C and D). In the SPH‐treated groups, renal architecture appeared relatively more preserved than that observed in the CP group. The SPH6 group showed more clearly defined glomeruli, Bowman’s capsules, and renal tubules (Figure 8E and F), whereas the SPH3 group showed generally preserved renal morphology, although tubular alterations and inflammatory cell infiltration remained observable (Figure 8G and H).

**FIGURE 8:**
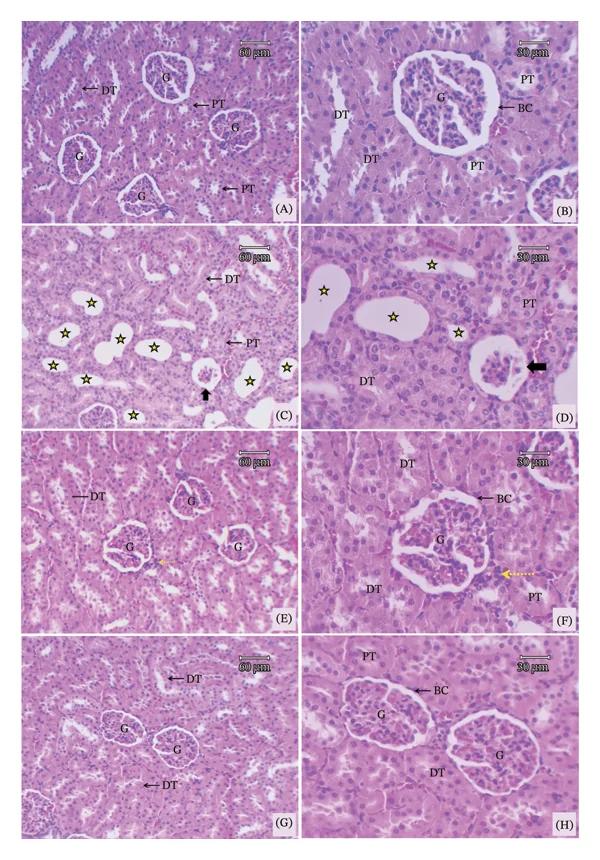
Histopathological features of renal tissues from cyclophosphamide‐treated rats receiving spirulina protein hydrolysate. Representative H&E‐stained kidney sections showing paired images at two magnifications for each experimental group: (A–B) NC, (C–D) CP, (E–F) SPH6, and (G–H) SPH3. Panels A, C, E, and G show lower‐magnification views of overall renal architecture, whereas panels B, D, F, and H show higher‐magnification views highlighting glomerular and tubular details. Yellow stars indicate dilated renal tubules; the yellow dotted arrow indicates inflammatory cell infiltration; and thick black arrows indicate shrunken glomeruli. Abbreviations: G, glomerulus; BC, Bowman’s capsule; PT, proximal convoluted tubule; DT, distal convoluted tubule. Scale bar = 60 μm in panels A, C, E, and G; scale bar = 30 μm in panels B, D, F, and H.

Histopathological examination of cardiac tissues showed relatively preserved myocardial architecture across all experimental groups, with regularly arranged cardiomyocytes and visible nuclei (Figure 9A). No obvious myocardial necrosis, inflammatory cell infiltration, or marked disruption of cardiac fibers was observed in the CP group compared with the NC group (Figure 9A and B). Similarly, cardiac sections from the SPH6 and SPH3 groups showed no marked morphological alterations (Figure 9C and D).

**FIGURE 9:**
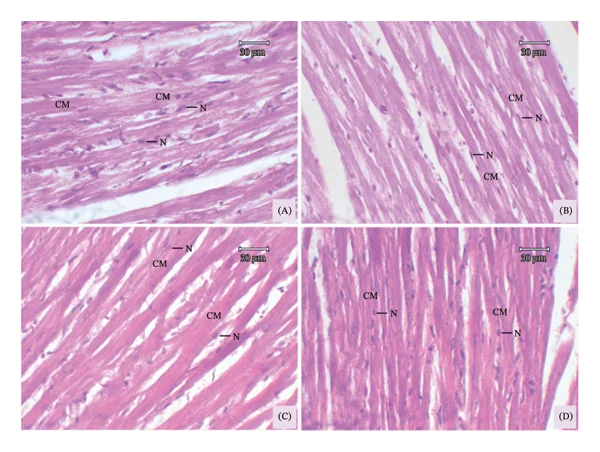
Histopathological features of cardiac tissues from cyclophosphamide‐treated rats receiving spirulina protein hydrolysate. Representative H&E‐stained sections of cardiac tissue showing (A) NC, (B) CP, (C) SPH6, and (D) SPH3. Abbreviations: CM, cardiomyocytes; N, nucleus of cardiomyocyte. Scale bar = 30 μm.

Histopathological examination of lung tissues from the NC group showed normal pulmonary architecture, with clearly visible bronchioles, open alveolar spaces, alveolar ducts, alveolar cells, and alveolar macrophages (Figure 10A). In contrast, lung sections from the CP group exhibited alveolar septal thickening and interstitial inflammatory cell infiltration (Figure 10B). In the SPH‐treated groups, pulmonary architecture appeared relatively more preserved than that observed in the CP group. The SPH6 group showed relatively more open alveolar spaces with less apparent interstitial inflammatory cell infiltration compared with the CP group (Figure 10C), whereas alveolar septal thickening and inflammatory cell infiltration remained observable in the SPH3 group (Figure 10D).

**FIGURE 10:**
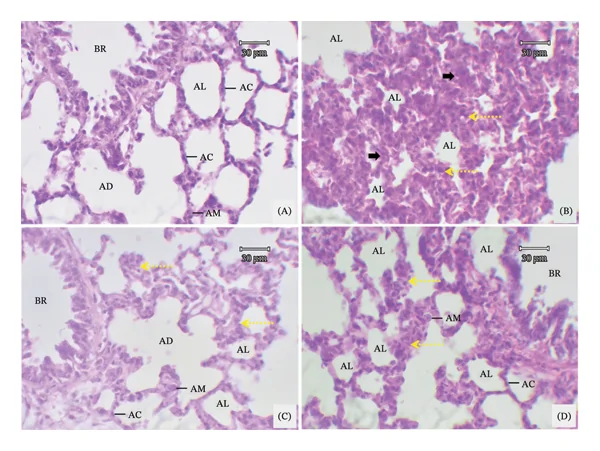
Histopathological features of lung tissues from cyclophosphamide‐treated rats receiving spirulina protein hydrolysate. Representative H&E‐stained lung sections showing (A) NC, (B) CP, (C) SPH6, and (D) SPH3. Thick black arrows indicate alveolar septal thickening; yellow dotted arrows indicate interstitial inflammatory cell infiltration. Abbreviations: BR, bronchiole; A, artery; AL, alveoli; AD, alveolar ducts; AC, alveolar cells; AM, alveolar macrophage. Scale bar = 30 μm.

Histopathological examination of testicular tissues revealed normal seminiferous tubule architecture in the NC group, with well‐organized spermatogenic cell layers, including spermatogonia, primary spermatocytes, spermatids, and spermatozoa within the tubular lumen (Figure 11A). In contrast, the CP group showed marked testicular damage, characterized by disruption of seminiferous tubule organization, exfoliation of germ cells into the lumen, vacuolization, and multinucleated giant cells (Figure 11B–D). In the SPH‐treated groups, seminiferous tubule architecture appeared relatively more preserved than that observed in the CP group. The SPH6 group showed more organized spermatogenic cell layers with less apparent germ cell degeneration (Figure 11E), whereas the SPH3 group showed relatively preserved seminiferous tubule architecture, although germ cell exfoliation remained observable (Figure 11F).

**FIGURE 11:**
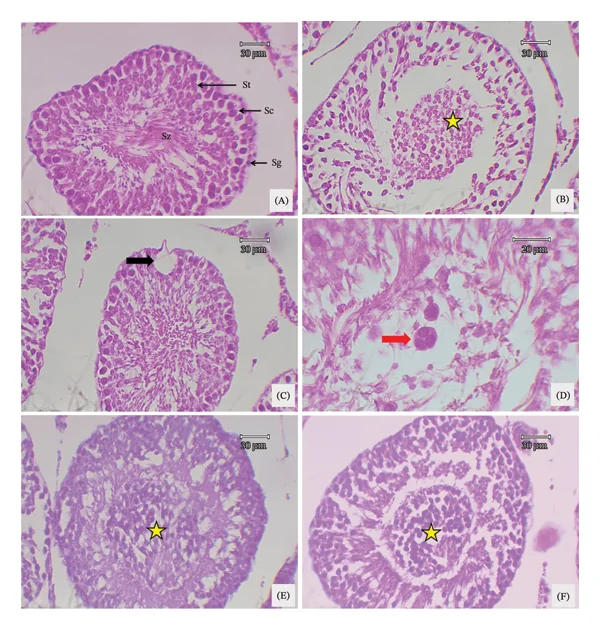
Testicular tissues of cyclophosphamide‐induced rats treated with spirulina protein hydrolysate. Representative H&E‐stained testicular sections showing (A) NC, (B–D) CP, (E) SPH6, and (F) SPH3. Yellow stars indicate exfoliated germ cells, a thick black arrow indicates vacuolization, and thick red arrow indicates a multinucleated giant cell. Areas of tissue separation were regarded as processing artifacts and were not interpreted as treatment‐related lesions. Abbreviations: Sg, primary spermatogonia; Sc, spermatocyte; St, spermatid; Sz, spermatozoa. Scale bar = 30 μm.

Histopathological examination of the epididymis in the NC group showed normal epididymal architecture, characterized by an intact epididymal epithelium, normal stromal structure, and abundant spermatozoa within the epididymal lumen (Figure 12A). In contrast, epididymal sections from the CP group exhibited clear cell hyperplasia, decreased spermatozoa within the lumen, germ cell sloughing, and areas with apparently lower luminal sperm density (Figure 12B and C). In the SPH‐treated groups, epididymal architecture appeared relatively more preserved than that observed in the CP group. The SPH6 group showed a relatively intact epididymal epithelium with greater apparent spermatozoa content within the lumen compared with the CP group (Figure 12D and E), whereas the SPH3 group showed relatively preserved epididymal architecture, although epithelial alterations and clear cell hyperplasia remained observable (Figure 12F).

**FIGURE 12:**
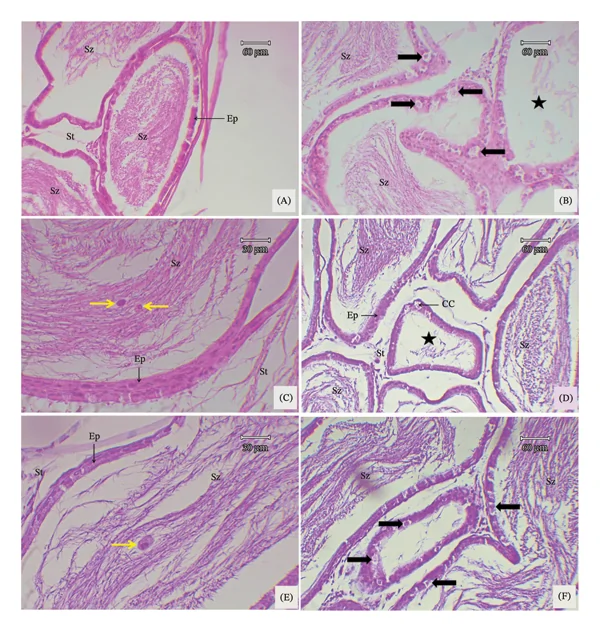
Histopathological features of epididymal tissues from cyclophosphamide‐treated rats receiving spirulina protein hydrolysate. Representative H&E‐stained sections of the cauda epididymis showing (A) NC, (B–C) CP, (D–E) SPH6, and (F) SPH3. Thick black arrows indicate clear cell hyperplasia; black stars indicate areas with apparently lower luminal sperm density; thin yellow arrows indicate germ cell sloughing. Areas of stromal separation were regarded as processing artifacts and were not interpreted as treatment‐related lesions. Abbreviations: Ep, epididymal epithelium; St, stroma; Sz, spermatozoa; cc, clear cell. Scale bar = 30 μm.

Histopathological examination of the seminal vesicle in the NC group showed normal tissue architecture, characterized by an intact seminal epithelium and luminal seminal secretion (Figure 13A). In contrast, the CP group exhibited epithelial alterations, primarily vacuolization of the seminal epithelium (Figure 13B). In the SPH‐treated groups, the seminal epithelium appeared relatively more preserved than that observed in the CP group, although mild epithelial vacuolization remained observable in both the SPH3 and SPH6 groups (Figure 13C and D).

**FIGURE 13:**
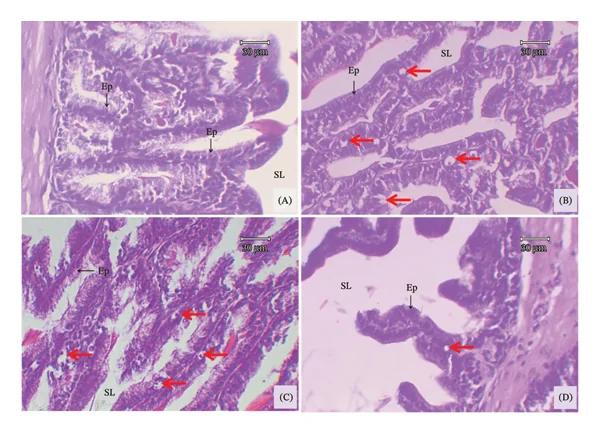
Histopathological features of seminal vesicle tissues from cyclophosphamide‐treated rats receiving spirulina protein hydrolysate. Representative H&E‐stained sections of seminal vesicle showing (A) NC, (B) CP, (C) SPH3, and (D) SPH6. Abbreviations: Ep, seminal epithelium; SL, seminal fluid. Thin red arrows indicate vacuolization. Scale bar = 30 μm.

Histopathological examination of prostate gland tissues from the NC group showed typical prostatic architecture, with an intact prostatic epithelium, normal stromal structure, and prostatic fluid within the glandular lumen (Figure 14A). In contrast, prostatic sections from the CP group exhibited epithelial vacuolization, leukocyte infiltration, apoptotic‐like cells, and sloughed cellular debris within the glandular lumen (Figure 14B and C). In the SPH‐treated groups, prostatic architecture appeared relatively more preserved than that observed in the CP group. The SPH6 group showed relatively preserved prostatic epithelial structure, although epithelial vacuolization remained observable (Figure 14D). The SPH3 group also showed relatively preserved prostatic architecture, although inflammatory cells and sloughed cellular debris remained observable (Figure 14E and F).

**FIGURE 14:**
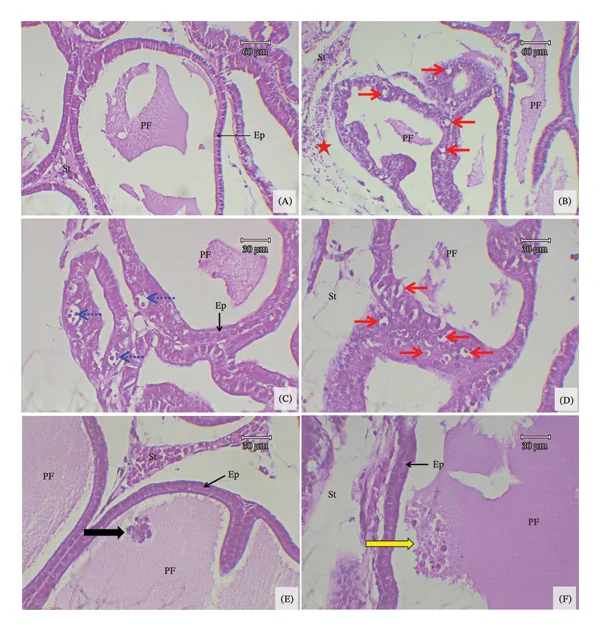
Histopathological features of prostate gland tissues from cyclophosphamide‐treated rats receiving spirulina protein hydrolysate. Representative H&E‐stained sections of the ventral lobe of the prostate gland showing (A) NC, (B–C) CP, (D) SPH6, and (E–F) SPH3. Thin red arrows indicate vacuolization; the red star indicates leukocyte infiltration; blue dotted arrows indicate apoptotic‐like cells; the thick yellow arrow indicates white blood cells; and the thick black arrow indicates sloughed cellular debris. Abbreviations: Ep, prostatic epithelium; PF, prostatic fluid; St, stroma. Scale bar = 30 μm.

## 4. Discussion

Presently, the use of food supplements derived from natural products to alleviate adverse effects of chemotherapy has attracted considerable attention. Given the vast diversity of bioactive compounds that nature has to offer, these products have emerged as promising sources of health‐promoting agents. Particularly for spirulina, the high interest is due to the diverse biological activities of natural products, including antioxidant and anti‐inflammatory properties [35], as well as their ability to enhance immune function [36] and even sensitize cancer cells to chemotherapeutic agents [37]. SPH has been regarded as safe, with recent reports on its bioactivities that could complement cancer chemotherapeutic activity. By identifying the most effective applications, it may play a crucial role in improving the quality of life for cancer patients undergoing treatment. Here, the SPH was assessed for its protective or mitigating effects against CP as a supportive dietary supplement.

CP‐treated rats showed a pronounced reduction in BW by the end of the experimental period, whereas the SPH‐treated groups maintained higher BWs than the CP group. Mean BW increased over the experimental period in both SPH‐treated groups, although their final BWs remained lower than that of the NC group. In contrast, food intake did not differ significantly among the experimental groups at any of the evaluated time points, despite numerically lower mean values in the CP group on Days 7 and 14. Water intake showed a more variable pattern across the experimental period; however, by Day 14, the CP group exhibited the lowest mean water intake among the groups. These findings suggest that SPH supplementation may be associated with attenuation of selected physiological alterations accompanying CP exposure, particularly the marked reduction in BW. Although antioxidant activity has been reported for SPHs [6], its contribution to the BW and intake responses observed in the present study remains uncertain. Relative organ weights showed no consistent pattern across the examined organs. In particular, changes in relative testicular and epididymal weights should be interpreted alongside final BW, sperm quality, and histopathological findings, because changes in the body‐weight denominator can influence these indices.

CP‐associated reproductive alterations were evident across several, but not all, endpoints in the present study. Relative testes and epididymis weights differed among the experimental groups, whereas the relative weights of the prostate gland and seminal vesicle did not differ significantly. Because relative organ weights are normalized to final BW, these findings should be interpreted together with changes in BW and other reproductive outcomes. Sperm concentration also did not differ significantly among groups. In contrast, sperm viability was significantly lower in the CP group than in the NC group, whereas both SPH‐treated groups showed significantly higher viability than the CP group, with values comparable to those of the NC group. Sperm motility showed a different response pattern: the CP group exhibited lower progressive motility than the NC group, while the SPH6 group showed higher progressive motility than the CP group, although it remained lower than that of the NC group. Progressive motility in the SPH3 group was comparable to that observed in the CP group. These findings indicate that the reproductive response associated with SPH treatment was endpoint‐specific, with more evident changes in sperm viability and selected motility parameters than in sperm concentration.

Previous studies have similarly reported CP‐associated reproductive toxicity, including alterations in sperm quality and testicular and epididymal structure [38, 39]. Afkhami‐Ardakani et al. [22] reported that whole spirulina supplementation in CP‐treated male Wistar rats was associated with higher epididymal sperm counts, improved tubule differentiation and spermiation indices, and fewer testicular histological abnormalities compared with CP alone, although several reproductive indices remained below control values. Direct comparison with the present study is limited because the spirulina preparation, CP regimen, treatment duration, and measured reproductive endpoints differed. In the present study, the higher sperm viability observed in both SPH‐treated groups and the higher progressive motility observed in the SPH6 group, together with relatively preserved reproductive tissue architecture in representative histological sections, support a potential role of SPH in modulating selected reproductive alterations associated with CP exposure. However, the absence of a significant effect on sperm concentration, the variation in responses among sperm parameters, and the qualitative nature of the histopathological assessment do not support a conclusion of generalized reproductive protection or restoration of fertility, which was not directly evaluated.

Serum biochemical findings showed that CP exposure was associated with significant alterations in selected hepatic, acid–base, and metabolic parameters rather than uniform changes across the entire biochemical profile. Compared with the NC group, the CP group showed lower ALB and ALB/GLB values and higher ALP, AST, and TBA levels, whereas TP and ALT did not differ significantly between the two groups. Both SPH‐treated groups showed higher ALB, TP, and ALB/GLB values and lower ALP, ALT, AST, and TBA levels than the CP group. These findings indicate that SPH administration was associated with favorable changes in selected serum parameters related to hepatic function and protein status, although they should not be interpreted as evidence of complete restoration of liver function. Previous studies have similarly reported improvements in serum protein‐related indices following spirulina supplementation under physiological stress [40].

The renal‐ and metabolism‐related findings were more heterogeneous. Serum CO_2_ was significantly lower in the CP group than in the NC group and was significantly higher in both SPH‐treated groups than in the CP group, which is consistent with a treatment‐associated change in systemic acid–base status. However, the renal indices did not show a uniform response: BUN was significantly lower than the CP group only in the SPH3 group, CRE did not differ significantly between the SPH‐treated and CP groups, and the urea/creatinine ratio remained significantly elevated relative to the NC group. Therefore, these findings do not support a generalized conclusion of renal functional recovery. In addition, TCH was significantly higher in both SPH‐treated groups than in the CP and NC groups, indicating that not all biochemical changes associated with SPH treatment were directed toward control values.

CP treatment was also associated with significantly lower serum AMY and CK activities than those observed in the NC group, whereas both SPH‐treated groups showed significantly higher activities than the CP group, with values statistically comparable to those of the NC group. These enzymes should nevertheless be interpreted cautiously because total serum AMY is not pancreas‐specific, while total CK does not distinguish among tissue‐specific isoenzymes [41–43]. Accordingly, the present findings are more appropriately interpreted as normalization of selected serum enzyme activities rather than direct evidence of pancreatic or muscular recovery. Organ‐specific biochemical markers, pancreas‐specific AMY, CK isoenzymes, and corresponding tissue‐level mechanistic measurements would be required to clarify the physiological basis of these changes.

The serum oxidative stress results showed a biomarker‐specific response rather than a uniform improvement in antioxidant status after SPH administration. GPx activity was significantly lower in the CP group than in the NC group, whereas both SPH‐treated groups showed significantly higher GPx activity than the CP group. GR activity was also significantly lower in the CP group than in the NC group. Both SPH‐treated groups showed higher GR activity than the CP group; however, the response differed between doses, as the SPH3 group was comparable to the NC group, whereas the SPH6 group remained significantly lower than the NC group. CAT activity also differed significantly among groups, with the SPH6 group showing higher activity than the CP and SPH3 groups, but not differing significantly from the NC group.

In contrast, MDA, GSH, and GST levels did not differ significantly among the experimental groups. SOD activity showed a distinct pattern: both the SPH6 and SPH3 groups had significantly lower SOD activity than the NC group, while the CP group did not differ significantly from either the NC or SPH6 group. Collectively, these results indicate that SPH effects were inconsistent across antioxidant and oxidative stress markers and do not support a generalized antioxidant effect. The primary treatment‐associated changes were observed in GPx, GR, and, at the higher SPH dose, CAT activity.

Previous studies provide biological context for these biomarker‐specific responses. Enzymatic hydrolysates derived from *S. platensis* proteins have demonstrated antioxidant capacity in vitro, including enhanced radical‐scavenging and reducing activities as well as reduced lipid peroxidation [6]. Similarly, low‐molecular‐weight peptides from *S. platensis* protein hydrolysate have been reported to reduce intracellular ROS and nitric oxide levels, limit MDA accumulation, preserve SOD activity, and improve cell viability under H_2_O_2_‐induced oxidative stress [44]. At the in vivo level, Abouzed et al. [45] found that whole spirulina supplementation modulated oxidative stress and antioxidant parameters, as well as inflammatory and apoptosis‐related responses, in a rat model of cisplatin‐induced renal injury. However, these studies differed from the present investigation in terms of spirulina preparation, experimental model, and measured endpoints. The peptide composition of the SPH preparation used in the present study was not characterized. Thus, while previous findings support the biological plausibility of antioxidant‐related activity, they do not establish that the observed changes in GPx, GR, and CAT were mediated by the same mechanisms. Identifying the active peptide components and investigating their effects on specific redox pathways are required to clarify the mechanisms underlying the present findings.

Histopathological examination demonstrated morphological alterations in multiple tissues following CP administration. The CP group showed hepatocellular necrosis, renal tubular dilation, glomerular shrinkage, inflammatory cell infiltration, alveolar septal thickening, disruption of seminiferous tubule organization, germ cell exfoliation, epithelial vacuolization, and apparently lower luminal sperm density in the epididymis. The apparently lower luminal sperm density observed in some epididymal sections should be interpreted as a localized qualitative histological finding, whereas sperm concentration was determined quantitatively from spermatozoa released from the entire cauda epididymis. This difference in sampling scale may explain why lower sperm density was apparent in some histological sections despite the absence of a significant difference in sperm concentration among the experimental groups. Sahu et al. [39] similarly reported multiorgan histopathological alterations, including dose‐dependent biochemical and histological abnormalities in the liver, kidney, brain, and testis of CP‐treated rats. However, their study utilized a single acute CP exposure and assessed tissues 24 h after administration, whereas the present study implemented a different dosing regimen. Therefore, the findings support CP‐associated tissue alterations; however, direct comparison of lesion severity and underlying mechanisms between the two studies should be made cautiously.

In contrast to the alterations observed in other organs, the present study detected no obvious morphological abnormalities in cardiac tissues. This finding contrasts with previous reports of CP‐associated cardiotoxicity. For instance, El‐Agamy et al. [46] observed pronounced cardiac histopathological lesions, as well as changes in cardiac injury markers, oxidative stress, inflammatory mediators, and apoptosis‐related markers, following a single intraperitoneal dose of CP at 200 mg/kg in male Wistar rats. Differences between their findings and those of the present study may be attributable to variations in dosing regimen, timing of tissue collection, tissue sampling, histopathological assessment, and biological variability. However, the absence of obvious cardiac lesions in the representative sections examined in the present study should not be interpreted as evidence of absent cardiac injury, as cardiac‐specific biomarkers and quantitative histopathological assessments were not conducted.

Representative sections from the SPH‐treated groups generally showed more preserved tissue architecture than those from the CP group across several organs, including the liver, kidney, lung, testis, epididymis, seminal vesicle, and prostate gland. Previous studies provide relevant biological context for these findings. Afkhami‐Ardakani et al. [22] demonstrated that whole *S. platensis* supplementation improved testicular histological and histomorphometric alterations in CP‐treated rats. However, direct comparison with the present study is limited due to differences in spirulina preparation, CP regimen, treatment duration, and measured endpoints. Specifically, the present study utilized a protein hydrolysate rather than whole spirulina.

In summary, the histological observations in the present study align with treatment‐associated preservation of tissue architecture in representative sections. However, these findings should not be considered quantitative evidence of a generalized protective or dose‐dependent effect. The histopathological assessment was qualitative and lacked blinded lesion scoring or quantitative morphometric analysis. Additionally, the study did not assess organ‐specific oxidative stress, inflammatory, and apoptotic markers. Future research should incorporate blinded semiquantitative histological scoring, quantitative morphometric analysis, and organ‐specific mechanistic markers to better determine the magnitude and mechanisms of these tissue‐associated responses.

Collectively, these findings indicate that administration of SPH was associated with favorable changes in selected physiological, reproductive, serum biochemical, antioxidant, and histopathological parameters in CP‐treated rats. Specifically, SPH treatment was associated with higher sperm viability, changes in selected sperm motility parameters, normalization of several serum biochemical indices, higher activities of selected antioxidant enzymes, and relatively preserved tissue architecture in representative histological sections. Nevertheless, responses varied across parameters, with several outcomes showing no statistically significant differences. Additionally, the histopathological evaluation was qualitative, and the study did not characterize the bioactive peptide constituents or organ‐specific mechanisms. Therefore, the present findings support further investigation of SPH as a potential supportive dietary intervention in the context of CP‐associated toxicity rather than establishing a generalized protective effect.

## 5. Conclusion

In conclusion, CP administration was associated with alterations in selected physiological, reproductive, serum biochemical, antioxidant, and histopathological parameters in male Wistar rats. SPH administration was associated with favorable changes in several of these endpoints, including higher sperm viability, changes in selected sperm motility parameters, normalization of selected serum biochemical indices, higher activities of specific antioxidant enzymes, and relatively preserved tissue architecture in representative histological sections. However, responses were not consistent across all measured parameters, and the histopathological findings were based on qualitative assessment.

These findings provide preliminary preclinical evidence that SPH may modulate selected alterations associated with CP exposure in rats. Further studies are required to characterize the bioactive peptide components, clarify the underlying mechanisms, incorporate quantitative tissue assessment and organ‐specific biomarkers, and evaluate the translational relevance of these findings before the potential of SPH as a supportive dietary intervention can be established.

## Author Contributions

Kittiya Phinyo: conceptualization, data curation, formal analysis, visualization, writing–original draft, writing–review and editing. Pachara Sattayawat: conceptualization, data curation, visualization, validation, writing–original draft, writing–review and editing. Jeeraporn Pekkoh: resources, supervision, project administration, funding acquisition, writing–review and editing. Kritsana Duangjan: methodology, investigation, formal analysis, validation, writing–review and editing. Supap Saenphet: methodology, investigation, resources, validation, writing–review and editing. Phongsadathon Aonmanee: investigation, validation, visualization, writing–review and editing. Sirasit Srinuanpan: visualization, writing–review and editing. Khomsan Ruangrit: visualization, writing–review and editing. Chayakorn Pumas: funding acquisition, visualization, writing–review and editing. Wararut Buncharoen: conceptualization, methodology, data curation, supervision, project administration, writing–review and editing.

## Funding

This project is also funded by the National Research Council of Thailand (NRCT): Contract No. N23H660024.

## Conflicts of Interest

The authors declare no conflicts of interest.

## Data Availability

All data supporting the findings of this study are included in the article.
